# Received Signal Strength Fingerprinting-Based Indoor Location Estimation Employing Machine Learning

**DOI:** 10.3390/s21134605

**Published:** 2021-07-05

**Authors:** Ladislav Polak, Stanislav Rozum, Martin Slanina, Tomas Bravenec, Tomas Fryza, Aggelos Pikrakis

**Affiliations:** 1 Department of Radio Electronics, Faculty of Electrical Engineering and Communication, Brno University of Technology, Technicka 3082/12, 616 00 Brno, Czech Republic; xrozum00@vutbr.cz (S.R.); slaninam@vutbr.cz (M.S.); xbrave01@vutbr.cz (T.B.); fryza@vutbr.cz (T.F.); 2 Department of Informatics, University of Piraeus, 185 34 Pireas, Greece; pikrakis@unipi.gr

**Keywords:** Bluetooth, fingerprinting, indoor navigation, machine learning

## Abstract

The fingerprinting technique is a popular approach to reveal location of persons, instruments or devices in an indoor environment. Typically based on signal strength measurement, a power level map is created first in the learning phase to align with measured values in the inference. Second, the location is determined by taking the point for which the recorded received power level is closest to the power level actually measured. The biggest limit of this technique is the reliability of power measurements, which may lack accuracy in many wireless systems. To this end, this work extends the power level measurement by using multiple anchors and multiple radio channels and, consequently, considers different approaches to aligning the actual measurements with the recorded values. The dataset is available online. This article focuses on the very popular radio technology Bluetooth Low Energy to explore the possible improvement of the system accuracy through different machine learning approaches. It shows how the accuracy–complexity trade-off influences the possible candidate algorithms on an example of three-channel Bluetooth received signal strength based fingerprinting in a one dimensional environment with four static anchors and in a two dimensional environment with the same set of anchors. We provide a literature survey to identify the machine learning algorithms applied in the literature to show that the studies available can not be compared directly. Then, we implement and analyze the performance of four most popular supervised learning techniques, namely *k* Nearest Neighbors, Support Vector Machines, Random Forest, and Artificial Neural Network. In our scenario, the most promising machine learning technique being the Random Forest with classification accuracy over 99%.

## 1. Introduction

Indoor localization, as defined in [1], is a process that is employed to obtain a user or device location in an indoor environment. While satellite-based global systems are available for outdoor positioning, their applicability in indoor positioning is largely limited by the high attenuation of signal penetrating buildings, numerous reflections and problematic satellite visibility which all lead to impossible or inaccurate positioning. Enabled by the progress in indoor localization technologies and related electronic devices [2], the demand for precise localization systems is in the spotlight of many industrial fields. The number of use cases where indoor tracking or localization of an object is essential has been continuously increasing [3]. To name a few, the applications may target healthcare (e.g., patient localization), office (e.g., access to printer based on location), or home environment (e.g., automatic light control). In the last decade, many works have investigated different indoor technologies, methods and systems. Several more detailed surveys are available in [1,4,5].

Among the most popular are technologies based on the signals of Local Area Networks (LANs or Wi-Fi) and Bluetooth Low Energy (BLE), a recent version of the Bluetooth standard. While LAN signals offer more stable measurements, BLE requires far less power. BLE has become one of the most frequently used short-range wireless communication systems for indoor localization purposes [6]. Utilizing the Bluetooth system for indoor localization has been a target of numerous studies including our own [7,8,9,10,11,12,13,14,15,16]. In these works, in general, one or combination of three basic techniques is employed. Received Signal Strength (RSS) [9] may be evaluated to determine location from signal propagation loss, Time of Arrival (ToA) may be calculated to reveal location from the duration of signal propagation, and Angle of Arrival (AoA) may be utilized to relate received signal direction with receiver location [1]. The RSS-based approach, which is easy and cheap to implement even with signals of limited bandwidth and omnidirectional antennas, is considered in this work.

The RSS-fingerprinting based localization is realized in two steps. At first, in the so-called offline (training) phase, a map of the indoor environment divided into cells with finite spatial resolution is created. Subsequently, the RSS values of the Radio Frequency (RF) signals received from fixed beacons are collected at positions of interest during a limited time period and assigned to the environment map to create an RSS-fingerprint map. Second, in the online (testing) phase, the actually monitored signal strength is compared to entries in the RSS-fingerprint map to estimate the location of an object. The performance of the fingerprint-based localization is determined not only by the precision of RSS measurement, but also by the algorithm used to align measurements in the testing phase with the offline RSS fingerprint map.

The RSS fingerprint-based indoor positioning has been received a lot of interest [9,10,11,12,13,17,18,19]. Recent studies have revealed that this positioning technology has good performance in complex environments (absence of Line of Sight (LoS) signal path), but overall computational cost is not negligible [20]. Most importantly, fluctuations in RSS values due to small scale fading and frequent changes in the characteristics of the indoor environment can significantly increase the positioning estimation error.

This paper addresses the challenge of aligning online RSS measurements with offline RSS fingerprint map by employing Machine Learning (ML) techniques. We aim to review the approaches to improving RSS-based location fingerprinting by means of ML found in the literature. To this end, we present a detailed survey of the work published so far. We find that the published work is not directly comparable since the setup for measurement and for the interpretation of results varies significantly. Thus, to provide also a quantitative comparison of the core techniques possible, we propose an indoor experimental scenario and create a database of RSS fingerprints and test measurements. With the use of these data, we compare how different learning algorithms can improve the location fingerprinting result. We start with the algorithms found in the literature, but include also more complex models such as Artificial Neural Networks (ANNs). The contribution can be summarized as follows:

- Provide a survey of state of the art in ML-based fingerprinting improvement (Section 2),
- Provide a re-usable dataset obtained from our measurement campaign (Section 3),
- Benchmark different ML-based classifiers with respect to their potential to improve location fingerprinting results (Section 4 and Section 5).

This paper is organized as follows. Introduction is followed by Section 2 in which an overview of the ML algorithms used to improve the RSS-based location fingerprinting is provided. The created BLE-based indoor localization system and the conducted indoor measurement campaign to create a re-usable dataset are described in Section 3. Performance study of different ML-based classifiers with purpose to improve fingerprinting-based location results is presented in Section 4 and Section 5. Finally, this work is briefly summarized and concluded in Section 6.

## 2. Related Work

The ML algorithms have been employed in several studies with the aim to improve the performance of localization techniques [21,22,23,24,25,26,27,28,29,30]. In the following text, we will make an overview of the state of the art to summarize the most popular techniques and their expected performance. In Table 1, we provide a summary of the ML techniques employed in the literature to improve the localization algorithms. The machine learning models described in the works cited below are in general well understood and widely used in the research community focused on machine learning. For more details on their principal ideas and operation, we refer an interested reader to a comprehensive introductory text available in [31] as an example.

In the following subsections, we split the related literature into two groups. Firstly, the methods focusing on improvement of the RSS fingerprinting approach through machine learning are presented. Secondly, we consider the methods in which other information is combined with RSS and processed jointly with a machine learning technique.

### 2.1. RSS-Based Fingerprinting Improvement

The authors of [21] present a fingerprinting experiment where the locations are represented as two dimensional coordinates on a rectangular corridor with sizes of 2.5 m × 4.5 m. The system uses remote server-deployed analysis of the signal strengths related to four Bluetooth beacon-transmitted signal and the positions are then aligned with a 0.45 m grid. The training of the algorithm used 5000 radio signal samples at each position and the performance was evaluated for up to 50 measured samples at each point. Two algorithms were applied for the position estimation—Support Vector Machine (SVM) and Logistic Regression. Although SVM was reported to achieve better performance (average error of 50 cm), it shall be noted that the results lack details on the training procedure, parameters, as well as total test dataset size.

In [32], the authors propose a system based on iBeacon RSS fingerprints. To improve the stability of RSS measurements, they employ Kalman filtering. Finally, a *k* Nearest Neighbors (*k*NN) classifier is used to estimate location using a fingerprint. In the experimental part of the work, location in an area of 14 m × 5.5 m with three beacons placed 6 m apart was determined with an average error of 0.71 m (maximal error 1.6 m). Although the authors present the result in average error distance, since they pose a classification problem, it is more important to state that 60% of the locations were assigned to correct points with the neighboring points being selected for misclassified locations. In a follow up work [22], the team has replaced the Kalman filter with near beacon selection to drop distant beacon signals as being prone to interference. Furthermore, a new feature, the device azimuth, is introduced as a result of magnetometer measurement. In this case, the reduced database size (distant beacons are not recorded in the fingerprint map) is traded for performance—the maximal error increased to 3 m.

A relatively large number of Bluetooth transmitters (Beacons) is used in [23], where the signal of 22 beacons is analyzed in order to obtain the location of a node. In total, 56 distinct location pints were allowed. The experiment was conducted in a room with dimensions of 10.5 m × 15.6 m located on the first floor in a 11-story building. The metric to align measurements with fingerprint samples is simple Sum of Squared Differences in the RSS indicator values. Even with this simple metric, more than 96% of locations were classified correctly.

Support Vector Machine and *k* Nearest Neighbor machine learning approaches have been used to decide on the location based on fingerprinted maps in [26]. In this work, the authors state that *k*NN performs superior to SVM with the best results achieved when the number of neighbors equal to five. Data were obtained in an experimental area with a total size of 4 m by 3 m. In a 1 × 1 m grid of analyzed locations, the mean squared error of location estimate in the best case was as high as 3.66 m (beacon distance up to 15 m).

Another study focused on an improved BLE fingerprinting based localization has been published in [24]. Here, the authors compare the performance of Random Forest and Naive Bayes in a relatively large area (whole building floor—rooms with different dimensions and a hall) with a dense deployment of 30 beacons. The study shows that multiple users can successfully be detected using the Random Forest classifier with the accuracy of 91%.

The work published in [25] considers a Deep Learning (DL) model for the location estimate based on BLE signal level in the context of hospital patient tracking. The results of this study are promising as the accuracy of 99.9% is reported in a case when Convolutional Neural Network and Artifical Neural Network are used in combination, utilizing not only the absolute signal levels, but also temporal information. The study uses a large number of anchors—153 devices placed on three floors of the hospital building, with a simple transmitting tag being localized from signals received at anchors.

A very simple artificial neural network architecture has been introduced for RSS based localization in [27]. Here, the authors use a Generalized Regression Neural Network (GRNN) which has a rather simple structure of a Multi-Layer network with a Radial Basis layer and a Linear layer. The algorithm is used to track a person moving in a rectangular area of dimensions 10 m × 15 m with four static anchors deployed close to area corners. The GRNN approach allows to achieve an average localization error of approximately 0.6 m. Combined with Unscented Kalman filtering, the average location error gets even one order smaller.

### 2.2. Fusion of RSS and Other Information

In [28], the authors have executed an experiment with a location grid of 1 × 1 m in a single room. The location is primarily determined from the received power level fingerprint, as measured from six Bluetooth anchors. Further, the estimate is improved through dead reckoning, i.e., analyzing the relative location changes over time. The two types of information are then used jointly as features for the ML. The study has employed two classification algorithms to process the measured signal strength levels—SVMs and Random Forests, with the latter showing better performance in terms of location error.

In [29], the authors present a whole system for Bluetooth RSS-based localization and navigation. Here, the user’s location is determined from received signal strength fingerprints by the *k*NN majority vote algorithm in the first phase. Then, a particle Markov model is applied to improve the location estimate by means of the trajectory, map information, and pedestrian behavior. The mean distance error has reached 0.32 m in case nine beacons are placed in an area of 7.1 m × 4.2 m. The results are significantly worse in case different devices were used to collect fingerprints and to perform the actual measurement.

Zhuang et al. in [30] propose a BLE-based localization system integrating a Polynomial Regression Model, RSS fingerprinting, outlier detection and Kalman filtering. The authors notice the difference in received signal level values in the three broadcasting channels of BLE and address this difference by creating a separate regression model and a separate fingerprint for each channel. Even for a static device, the received power levels are reported to vary significantly—up to the order of 10 dB—which makes the location estimate difficult to derive. To this end, an extended Kalman filter is used in order to stabilize measurement results for a given location. With this setup and a sparse deployment of beacons (18 m apart), the location error is below 3.88 m for 90% of the cases. With a denser deployment (beacons 9 m apart), the error is below 2.56 m for 90% of the cases.

## 3. Indoor Measurement Campaign

To create a comprehensive database of RSS fingerprints, extensive indoor measurements have to be performed. To this end, we have set up a BLE-based indoor localization system using single-input multiple-output (SIMO) approach. Its basic concept has been introduced in [12] and a part of the dataset used in this work has been obtained from our previous measurements [12]. In this section, the whole measurement campaign and the indoor environment are described in detail.

### 3.1. Remote and Self-Positioning

Before we describe our system, let us briefly introduce two main concepts of indoor positioning, namely remote-positioning and self-positioning systems [33]. The difference lies in the roles of fixed and moving elements of the system.

**Figure 1:**
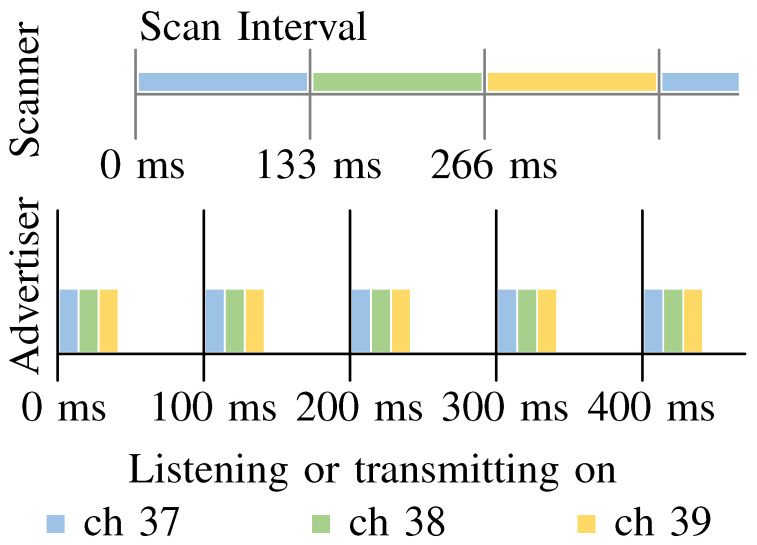
Timing of advertisement transmission and reception. The BLE module sends three identical consecutive advertising packets on different advertisement channels on every advertising event. The scanner receives only one report depending on the listening channel. It can actively listen for the whole Scan Interval or only a part of it. Time marks are relative to the Advertiser or Scanner and as such they are intentionally misaligned to illustrate the real-world situations, where the BLE communication is not synchronized.

In the remote positioning mode, the fixed anchors are listening for reports and movable tags transmit reports. This approach benefits from the option to have more than one receiving antenna because the space limitation and power consumption are not a significant issue. Hence, it is possible to collect multiple RSS samples for a single advertisement (see Figure 1). For example, each antenna (connected to a dedicated receiver) may listen on a specific channel or be spatially placed for diversity. Ideally, the multiple uncorrelated samples offer more ways to improve the accuracy of a positioning system, especially for moving targets. Additionally, a remote positioning system enables to track not only smartphones but also very simple tags.

Since during the calm work hours, it is not a problem to catch reports from more than 20 tags in an indoor environment, the system communication layer must ensure high throughput, low latency and highly reliable connection from anchors to the processing server. Demanding system communication and the need for back-channel in situations where the resolved position needs to be sent back to the tag for navigation purposes are the downsides of this mode. Another disadvantage lies in the increasing RF noise with a rising number of tags.

On the other hand, in the case of the self-positioning mode, where fixed anchors transmit advertisements and tags receive them, the system communication layer has almost no requirements. A typical BLE receiver can listen on a single channel at a time, which means that the devices receive reports from all anchors on the same channel during the time scan interval (see Figure 1). Further, the anchors cannot transmit the reports at the same time. Otherwise, the advertisement reports can interfere with each other and be lost. This decreases the number of collected RSS samples and limits channel diversity, notably for moving targets. Although it may seem that the self-positioning system does not share the disadvantage of increasing RF noise with a rising number of tags, it is not entirely true. Tags such as smartphones, wearables and other personal devices transmit BLE advertisements on their own. For example, when the user wants to locate himself, their smartphone collects the RSS samples from anchors but also from their watch, which may introduce interference. A remote positioning system can receive reports from both watch and smartphone, but otherwise, it is passive (no reports from anchors).

Therefore, the impact of active BLE devices on RF noise is about the same as in the remote positioning system.

### 3.2. Experimental Setup

**Figure 2:**
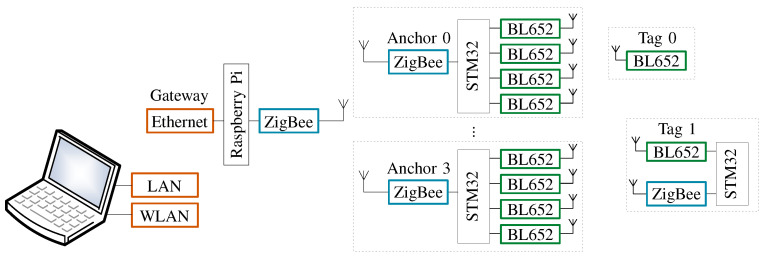
Block diagram of the BLE-based localization system using SIMO approach (based on [12]).

Block diagram of the proposed BLE-based localization system is shown in Figure 2. From left to right, a personal computer (PC) is used to process and log the RSS samples (the data are processed offline later). For data logging (the source code for data logging is available for download here: https://github.com/Standa-R/BlePositioningSystem, accessed on 4 July 2021), the msi GT72 2QD Dominator notebook with Windows 10 operating system and Microsoft Visual Studio integrated development environment was used. Raspberry Pi B+ (acting as a gateway to Ethernet) exposes the application programming interface (API) to control and collect data from anchors. The ZigBee network established with Silicon Labs ETRX357 ZigBee modules [34] secures the system communication layer, which is responsible for transferring data from anchors to Raspberry Pi. The positioning layer is built from Laird Connectivity BL652 BLE modules [35]. Apart from the gateway, all parts of the system are entirely tetherless, which assures high portability and short setup time for the measurement.

The ZigBee module acting as a coordinator connects Raspberry Pi B+ to the ZigBee network. To avoid a direct connection to the pins of the processor, the ZigBee module interacts with Raspberry via FTDI USB/UART converter.

To support easy setup and functionality of the ZigBee network in harsh RF environment, the RF channel used to transfer data is automatically selected from the IEEE 802.15.4 frequency band (2.4 GHz) by the ZigBee module. The ZigBee channels 15 and 26 (center frequencies 2.425 and 2.480 GHz) are excluded from the automatic channel selection list due to the risk of interference with BLE advertisement channels 38 and 39 (center frequencies 2.426 and 2.480 GHz). Low data rate is one of the main drawbacks of the ZigBee network. For the remote positioning mode, the bottleneck is even more narrowed by the maximum UART baud rate of the ZigBee ETRX357 module, which is 115.2 kbps. We assume that the tags transmit advertisement every 100 ms (see Figure 1). Therefore, we can estimate the absolute maximum number of tags tracked by the system at a time. Each advertisement report transmitted by a tag generates an anchor report, which contains 29 bytes of useful information (protocol header, advertiser ID, timestamp, 4 × RSS, 4 × channel). However, due to the protocol overhead of the ZigBee module, it transmits 49 bytes to Raspberry Pi B+ via UART. The system contains four anchors, which means that each advertisement report requires 200 bytes transmitted via UART. Hence, while neglecting RF collisions, the coordinator can theoretically accept a maximum of 7 tags. Practically, the system with three tags already experiences high latency, out of order samples (several seconds old samples), and losing of samples. Therefore, a configurable address filter discarding any report with advertisement address different from the allowed list was implemented in the anchors. This solution, however, causes a situation when the ZigBee-based transport layer is not suitable for cases where a high refresh rate is required. Nevertheless, the realized system is portable and excels with its straightforward implementation in an indoor environment with reliable reproducibility of the measurements, which are the most important parameters for our experimental purposes.

The anchor firmware runs on STM32F091CCT [36], the smallest 32-bit MCU from STMicroelectronics with six UART modules. Four UART modules are used for BL652, one for ZigBee and one for debug. Four fixed anchors, each fitted with four BL652 modules and one Silicon Labs ETRX357 ZigBee module (see Figure 2) perform localization-based communication (measuring). Since BLE652 modules are software controllable, the system supports both remote (anchors are listening while tags periodically transmit reports) and self-positioning (anchors periodically transmit reports and tags are listening) modes. The anchor has four omnidirectional dipole antennas mounted in line with a spacing of λ/4, where λ corresponds to wavelength in the center of the 2.4 GHz ISM band, i.e., 2440 MHz (channel 17). Depending on the antennas being either straight (horizontal polarization) or bent (vertical polarization), the antenna spacing can be either λ/4 or λ/2. In this work, the half-wavelength spacing was used. Each anchor is powered from a single Li-ion 18650 cell, which can provide power for more than 24 h on a single charge even under the full load. To minimize ZigBee traffic, the anchor sends a single packet per BLE report. Due to the fact that each BL652 module listens on different RF channels at a time (see Figure 1) and random processing delays are experienced, the BL652 modules (antennas) do not receive the report from the BLE advertising event of the transmitting tag at the same time. Therefore, the microcontroller buffers the reports according to the advertiser’s ID and schedules them for ZigBee transmission once all four measurements are available. When not all four reports have been received in a four-millisecond time window, the anchor assumes that the BLE module was not able to receive the report and transmits the measured RSS values available from other antennas. Anchors extend each report by a timestamp, which enables further processing units to relate the reports from all anchors to each advertisement event. Considering a minimum interval between two advertisement events (20 ms), the maximum synchronization error was set to 10 ms. The time reference of the anchors is synchronized periodically.

The proposed system can work with any BLE tag, e.g., custom tag (Tag 1), smartphones or wearable devices. In Figure 2, Tag 0 represents the simplest and smallest tag. It has no link to the system communication layer, which limits the usage to the remote-positioning mode. Hence, it only transmits BLE reports while the anchors measure and send RSS samples for processing. These tags can be placed, for instance, on the equipment we want to track. On the contrary, Tag 1 employs the ZigBee module (connection to system communication layer), which enables its configuration and data collection via the API. Therefore, Tag 1 can work in both remote and self-positioning modes. In this work, we used Tag 1 equipped with an omnidirectional dipole antenna [37] for BLE module. Further, it is connected to the ZigBee network via the ZigBee module, which uses the second omnidirectional dipole antenna. In the case of remote-positioning mode, the tag periodically transmits BLE reports and only listens for messages from the ZigBee network. On the other hand, in self-positioning mode the tag listens for reports and sends the measured RSS (from BLE channels) to the gateway over ZigBee network. In the following analysis, we focus on the results obtained in the remote positioning mode.

### 3.3. Indoor Environment

**Figure 3:**
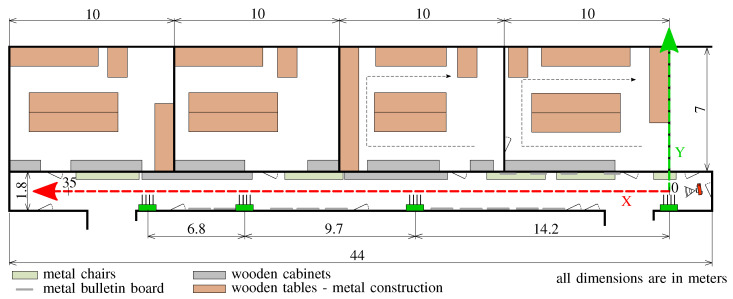
Measurement scenario and floor plan. Green boxes denote the anchors. The height of the anchors is 2.2 m above the floor level, the height of tags is 1.0 m. Scale of X axis and Y axis is different. Room dimensions are approximate.

**Figure 4:**
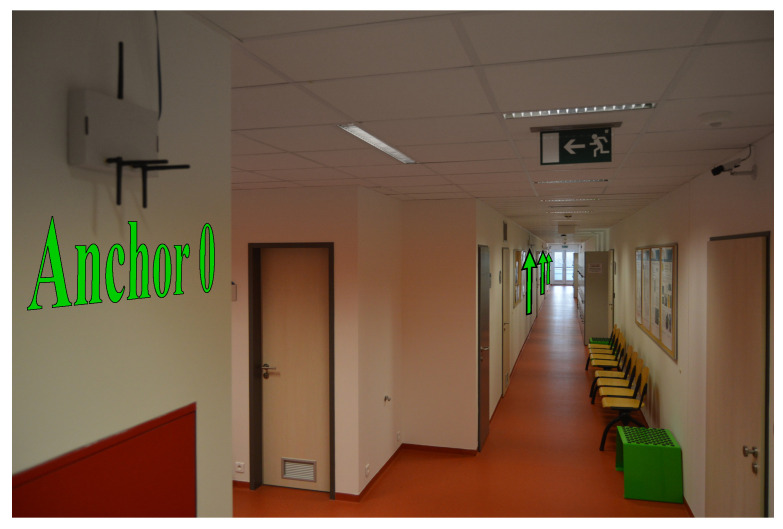
The installed anchors [12]. The photo is taken from the marked position (red camera in Figure 3), Anchor 0 and green arrows indicate anchors’ position.

Measurement campaigns were conducted in the building of Brno University of Technology (BUT), at the Department of Radio Electronics (DREL). The measurement scenario and floor plan are illustrated in Figure 3. The complete localization system was installed in a narrow corridor with dimensions 44 × 1.8 × 2.7 m, located on the seventh floor of DREL. The floor is made from concrete, walls are lined with plasterboard and the ceiling material is mineral wool with paint finish. Wooden cabinets and metal chairs are located on the right side of the floor, whereas the left side is without obstructions (see Figure 4). Altogether seven laboratories are located on this floor with an approximate dimension of 7 × 10 m each. They have wardrobes, operable windows, several chairs, two tables and one desk. Two of the laboratories have been used to collect measurements in our setup as shown by the black dashed arrow in the right-hand side of Figure 3.

### 3.4. Measurement Scenario

As seen in Figure 3 and Figure 4, the reference point [0, 0] m of the two-dimensional coordinate system is placed at the position of Anchor 0 at the height of 1 m above the floor level, which is often considered as the common height for moving tags. In general, the accuracy of indoor localization is also influenced by the placement of anchors. Many times, it is a trade-off between the mounting possibilities and the characteristics of indoor environment. Based on the considered indoor environment conditions, anchors with ID from 0 to 3 were installed 2.2 m above floor level. In the two-dimensional system, they have coordinates [0, 0], [14.2, 0], [23.9, 0] and [30.7, 0] m, respectively. It means that anchors are above the tags and above the height of the people. To achieve the lowest possible fluctuations of the RSS values during the measurements, the tag was in a constant height of 1 m and the movement of people was minimal.

The measurement with a step of 0.5 m in the X and Y axes (see Figure 3) was conducted in the corridor from 0 to 35 m and 0.4 to 1.4 m, respectively. Each measurement lasted 72 s for remote positioning and 140 s for self-positioning. There were collected about 670 records at each position (in total more than 140,000 records), where each record contains RSS samples from four antennas from four anchors. Since the tag in the self-positioning mode has only one receiver and one antenna, it can collect only one sample at a time. Therefore, it takes three scan intervals (see Figure 1) to collect samples on each channel from the anchors. During these three intervals, each antenna sends three BLE advertisements. The tag buffers the samples, and ideally, after the three scan intervals, it should send three messages for processing in the same format as the anchors send in remote-positioning. However, due to interference and radio tuning, sometimes, the report was missing. Thus, the measurement time was doubled, and finally, measurement samples were trimmed. That way, samples were collected on different channels, but at different times. In the remote-positioning mode, the BLE report does not contain any user data, only ID of tags (it is a random number). In the case of self-positioning mode, the message contains only the ID of antenna and anchors. In a transmission channel with time-independent fading, it is a usable approach for the fixed tags. For tags in motion, the movement speed and advertising period are the limiting factors. For measurement conducted in the corridor the tag was always in the Line-of-Sight (LoS).

Further measurements were carried out in laboratories which is a Non-Line of Sight (NLoS) condition for the signal. In Figure 3, the measurement path is marked by black arrows. The step was again 0.5 m, but only the places free to walk (i.e., not obstructed by furniture) were covered.

### 3.5. Dataset

The complete measurement results are provided as an open dataset to be re-used by the research community. The complete data are contained in a comma-separated values (.csv) text file, which has been preprocessed in the following steps:

- The raw measurement, collected as the received signal levels at anchors in the remote positioning mode, contain, for each anchor, four channel numbers and four corresponding RSS levels, as measured by four BLE modules (onboard each anchor). At this point, it is not assured that RSS levels at all three advertisement channels are recorded and that all values are valid. In the self-positioning mode, one measurement contains the same set of values, although taken in the opposite direction.
- All values where RSS level of −110 dBm was recorded are considered to be missing measurements as at this level the receiver fails to measure the actual received power level.
- In order to ensure there are no missing values, records from two consecutive measurements are taken as one sample. For each anchor, the first valid measurement is recorded in the sample and the remaining measurements are discarded (e.g., measurements from further antennas). This allows to clean the data set in such a way that no missing values appear in the data.

In the end, for each tag position, there are three RSS signal levels collected at three advertisement channels at/from each anchor. This means that each position is characterized by twelve (three samples multiplied by four anchors) measured values, defining the feature space for the machine learning algorithms.

The dataset is available for download from: https://github.com/slaninam/Loc1D/tree/master/data_csv (accessed on 4 July 2021).

## 4. One-Dimensional Positioning

**Figure 5:**
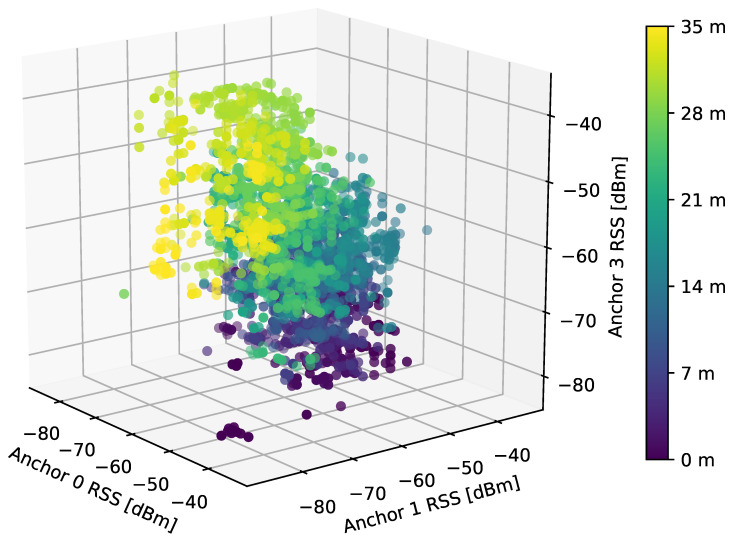
A visualisation of RSS values received from three anchors in a three-dimensional space, channel 37 only. The color of the points represents the location distance. For simplicity, only 20% of data has been used in the plot.

In the one-dimensional positioning, we aim to estimate the location along a single axis. To this end, four anchors parallel to this axis are used in the RSS power measurement. In the measurement data, we select a subset of the center of the corridor—the *Y* axis coordinate in this case is limited to 0.9 m. In Figure 5, we visualize the measured RSS when taking into account three of the four anchors, in order to limit ourselves to a three-dimensional space which can well be captured in a picture. The color of the points represents the distance along the considered axis. Obviously, the points with the same color tend to appear in similar locations in the three-dimensional space, which poses a promising starting point for any classification algorithm.

**Figure 6:**
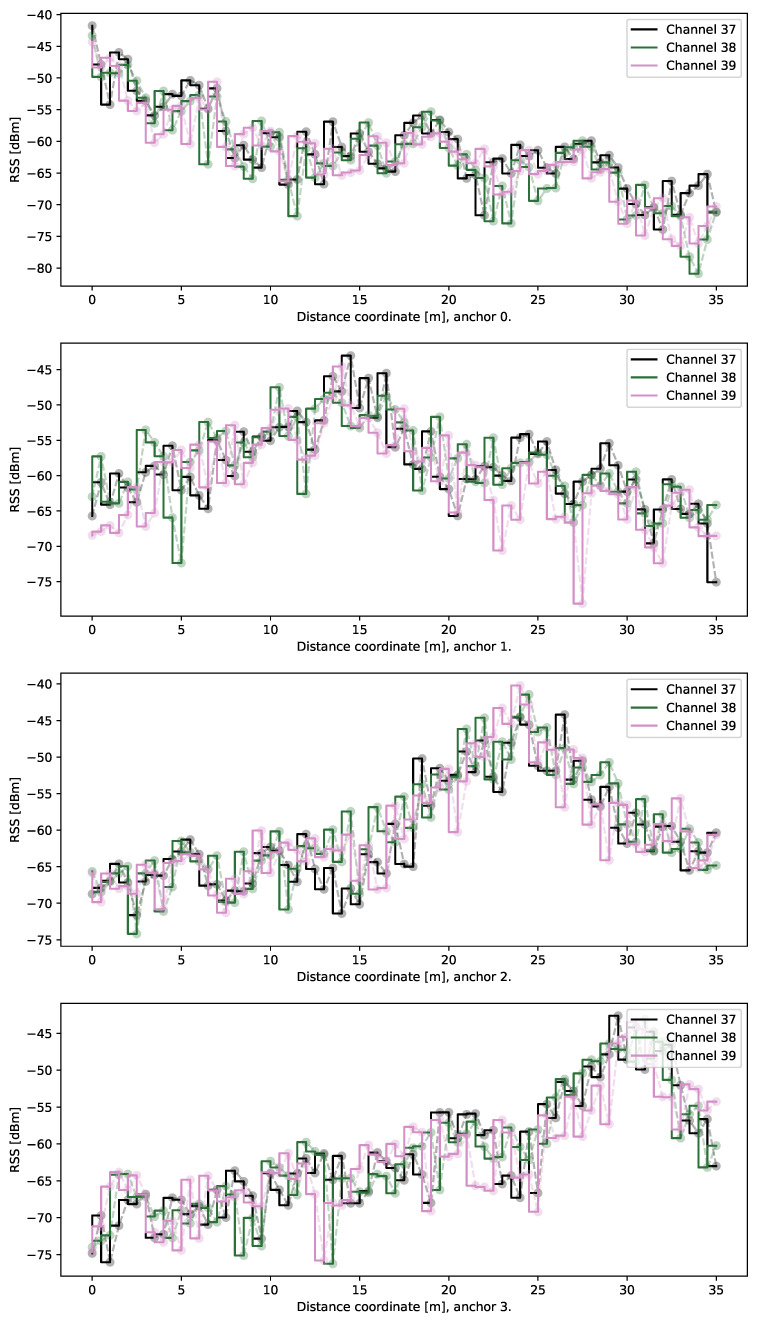
Mean values of received signal strengths at four anchor location when the transmitter is the moving tag. For each location, the mean of all received measurement samples is displayed. Peaks of RSS are visible for all advertisement channels for distances from 0 to 35 m.

To illustrate the situation further, we present the mean RSS values for signals belonging to each anchor and three channel frequencies in Figure 6. Although the overall trend of RSS values being larger in areas close to the respective anchors, it shall be noted that the stability of the measurements is limited—the mean values tend to fluctuate in neighboring locations which prevents the position from being determined from signal levels directly. While the mean values are displayed in the figure, we have observed a very similar behavior also for the median values of RSS.

In order to evaluate the performance of different algorithms in the localization problem, we have split the whole dataset into the training set, and the test set. The split is done in such a way that from the measurements obtained at each position, the first 80% samples in time are allocated to the training set, while the remaining 20% samples are allocated to the test set. For the split, the samples are ordered by time stamps, however the time stamps are removed from both sets after the split to prevent data leakage.

**Figure 7:**
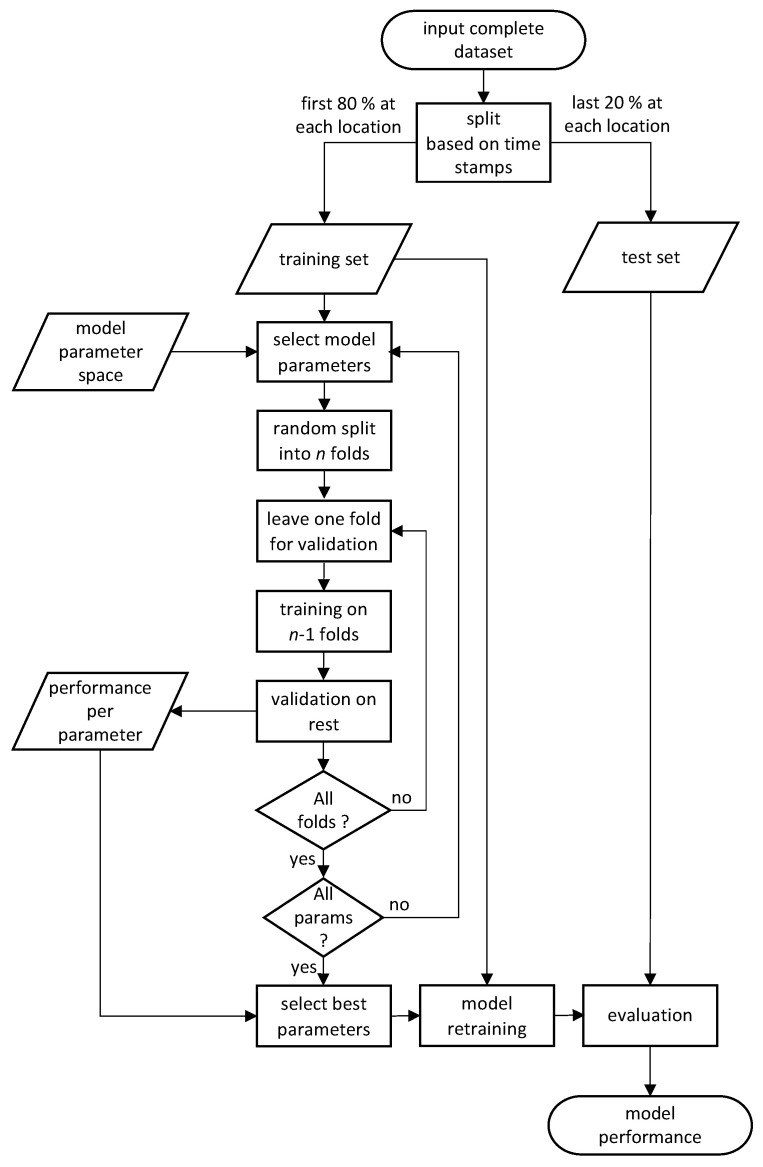
Flowchart of the model training and testing procedure. There are two phases of the training. At first, we look for the best model parameters on the training data (with no knowledge of the test data) by splitting the training data into *n* folds, then training on n−1 folds and validating on the rest. This way, for example, we find the number of neighbors for the best performance in the kNN model (e.g., we find that k=1). Then, when we are ready to select the best model parameters—we create the final model on the whole training set with the prerequisite that k=1. Only this final training is then used to evaluate the performance on the test set.

The training of the model is done on the training set only, with no information about the testing set available to the training algorithm. Since in general the models considered in this work allow for different hyperparameter values (e.g., the number of neighbors *k* to consider in the *k* Nearest Neighbors classifier or the number of neurons per layer in the Multi Layer Perceptron classifier), we have used the *n*-fold cross validation approach to determine the appropriate model parameters. In principle, the training set is randomly split into *n* groups of samples (folds) of equal sizes. All models have been trained with n=5 folds in the cross-validation search for model parameters. The value has been selected as a trade-off between the computational complexity (low number of folds leads to faster model selection) and prediction error (high number of folds leads to low prediction error) as discussed in [38,39]. Since the cross validation in this case is only used for primary selection of the model to be trained, the prediction error is not critical and the relatively low number of folds gives satisfactory results. Then, n−1 folds are used for training and the remaining fold is used for validation. This procedure is repeated *n* times so that each fold is used for validation once. In the end, the average performance of the model (accuracy achieved on the validation fold) across the *n* runs is recorded. After the complete desired parameter space of the model is explored and the validation outputs are obtained, the model structure performing best in cross-validation is used for final training of the model on the whole training set. This final model is then evaluated on the test set to obtain the model performance in terms of accuracy. The whole procedure is displayed in detail in Figure 7. For training the models, the Amazon Web Services (AWS) platform was used, utilizing an Elastic Computing Cloud (EC2) instance of the g2.8xlarge type selected with emphasis on system memory. The instance was running the Anaconda with Python 3 (x86_64) Amazon Machine Image so no special software installation was required. In the whole study, we have used Python programming language and its related libraries. The core of the training and evaluation is using scikit-learn (https://scikit-learn.org, accessed on 4 July 2021). More details can be found on: https://github.com/slaninam/Loc1D (accessed on 4 July 2021).

The time correlation of RSS samples is a phenomenon which has been studied in [40]. The authors have shown that the samples obtained for the same location are correlated in general, irrespective of the time difference between the collection of separate samples—they report that the mean and variance of the RSS in one location remains the same over time and the auto-covariance function of the RSS in one location has the same shape for separate time series. This effect does not only allow the machine learning approach to be employed for location estimation, but further investigation of the RSS correlation is reported to have the potential to improve classical localization algorithms. The temporal variation of RSS is also considered in [41], where the authors state that the most temporal fluctuations of the RSS are caused by moving objects, varying electromagnetic wave landscape, directionality of antennas and RF interference. A combination of fingerprints from several access points brings the benefits of having the mean differences of RSS more stable over a longer period of time.

The target values are represented as categorical variables, corresponding to the discrete spatial positions at which the measurements have been taken. In total, there are 70 categories corresponding to position on a 35 m long corridor with 0.5 m spacing.

We evaluate the performance of the algorithms through accuracy, i.e., the proportion of samples for which the correct location was estimated (in the ideal case, accuracy would be 1.0 meaning the location for 100% samples is estimated correctly). In the implementation, we have used the scikit-learn [42] library.

### 4.1. kNN Classifier

The *k* Nearest Neighbors classifier is a very popular approach in machine learning-based location fingerprinting as seen in Table 1. The idea behind this technique is to (i) remember the whole set of samples in the training phase and (ii) find the majority vote among *k* nearest samples in the training set to determine the estimate in the test sample.

In the design of such classifier, the following needs to be considered:

- The entire training set needs to be stored, which can lead to large memory requirements for bigger datasets.
- The metric to measure the distance from neighbors needs to be selected. A common choice is the Minkowski metric, defined for two points X,Y in the *n*-dimensional space as:
  (1) $$D\left(X,Y\right)={\left(\sum_{i=1}^{n}{\left|x_{i}-y_{i}\right|}^{p}\right)}^{\frac{1}{p}},$$
  in the simplest cases with the parameter p=1 leading to Manhattan distance and with p=2 leading to Euclidean distance.
- The number of neighbors to be considered is a design choice for the algorithm and depends on the characteristics of the dataset. In general, higher values of *k* lead to smoother decision boundaries, while smaller numbers of *k* capture data variations more faithfully.

In order to test the performance of *k*NN on our dataset, we vary the parameter *p* between 1 and 20 and select the Euclidean distance as the distance metric. In line with the flowchart in Figure 7, we use 80% of the samples for training and the remaining 20% for testing. To tune the classifier hyperparameter, i.e., the number of neighbors in this case, we perform 5-fold cross-validation on the training set. The classifier performing best is then tested with the test set.

**Figure 8:**
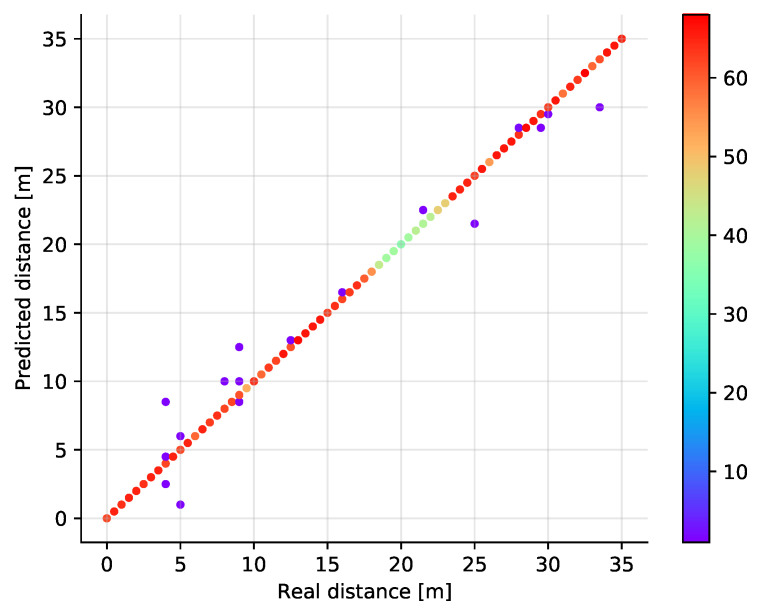
Scatter plot showing the predicted versus the real distances (test set) in a single dimension for the *k*NN classifier with *k* = 1. The color of the points represents the number of points at a given location.

**Figure 9:**
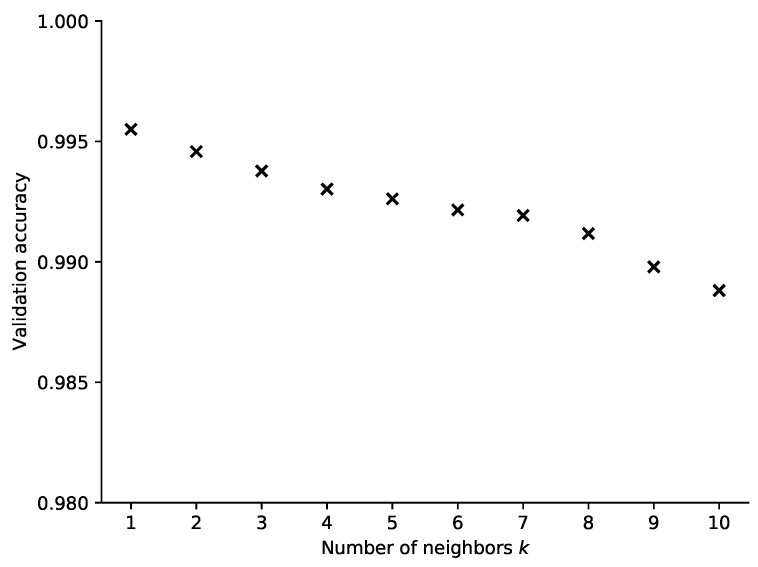
Mean accuracy of prediction achieved on the validation set for a varying number of neigbors in the *k*NN classifier.

The classification results are shown in Figure 8 where the real and estimated positions are plotted in a scatter plot diagram. The color of the points corresponds to the number of samples at a given position, showing that the off-diagonal points are rather isolated observations. It is also visible that some positions have less samples in total. This is a result of the measurement data post-processing. At some positions (distance around 20 m), it turns out that in relatively many measurements invalid values appear. Thus, after filtering, fewer valid samples remain. Although this may be unfair to the model, it reflects well the real life situation when also the system needs to handle invalid measurements. The best validation results have been achieved in case the number of neighbors is set to just one. Then, when the number of neighbors is increasing, the performance of the algorithm deteriorates gradually (see Figure 9).

The accuracy of the positioning algorithm, i.e., the number of distances classified correctly in the test set, was more than 99.5%. The mean and maximal errors in terms of predicted distance are shown in Table 2. The most distant prediction corresponds to the error of 4.5 m. Since a majority of the samples are classified correctly, the mean absolute error is in the order of millimeters. Still, we shall be aware that we are solving a classification problem, so the real distance error is discrete—either zero or multiples of 0.5 m in each sample.

### 4.2. Support Vector Machines

Another rather popular approach to the fingerprint classification is the SVM algorithm. The idea behind this is finding the separating hyperplane, such that the distance between the hyperplane and the closest samples belonging of distinct classes is maximized. Naturally, the dimensionality of the hyperplane depends on the number of features in the data set, and the description of the problem will be rather simple in case the number of features is low.

In general the Support Vector Machine classifier is searching for vectors of weights $w\in R^{p}$ and biases b∈R based on the training vectors $x_{i}\in R^{p},i=1,\ldots ,n$ such that the prediction given by $\mathrm{sign}(w^{T}\phi \left(x\right)+b)$ classifying the inputs to two classes $y\in {1,-1}^{n}$ is correct for most samples. The problem can be formulated as

(2) $$\min\;\frac{1}{2}w^{T}w+C\sum_{i=1}^{n}\xi_{i},$$

subject to

$$y_{i}\left(w^{T}\phi \left(x_{i}\right)+b\right)\geq 1-\xi_{i},$$

$$\xi_{i}\geq 0,i=1,\ldots ,n.$$

In this expression, the search for the maximum classification margin is represented by the minimization of ${\left||w|\right|}^{2}=w^{T}w$, while the penalty term allows for classification in cases where the problem is not perfectly separable with a hyperplane. Here, the parameter *C* controls the strength of the penalty term, i.e., adjusts regularization in the training process. The term $\phi (x_{i})$ represents the images of input vectors in feature space (dependent on the kernel function) and ξ is the loss term.

**Figure 10:**
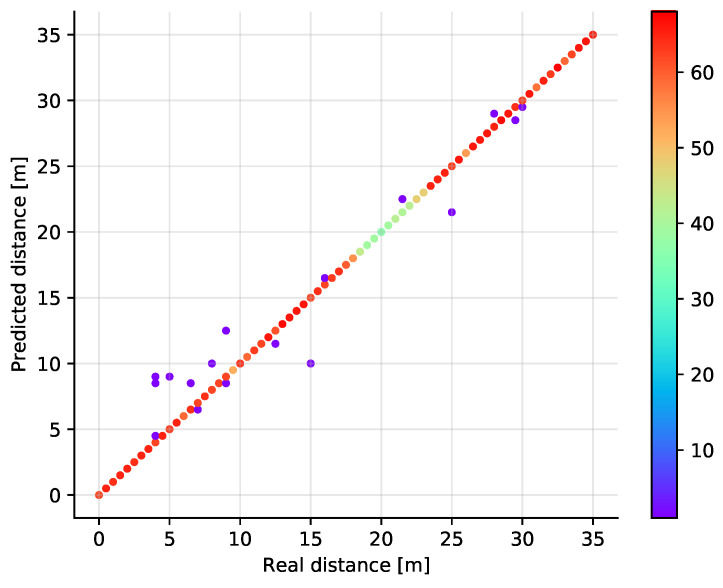
Scatter plot showing the predicted versus the real distances in a single dimension for the SVM classifier with C=20.

**Figure 11:**
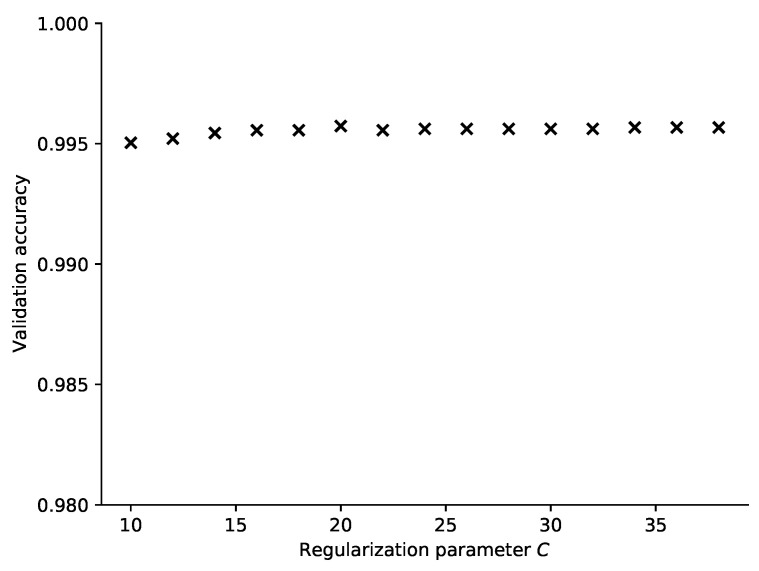
Accuracy of prediction achieved for a varying regularization parameter *C* in the SVM classifier.

We have trained the SVM classifier using the Radial Basis Function kernel with a variable regularization parameter to see how good it performs on our dataset. Since the SVM classifier is basically a binary classifier, the extension to multiple classes has been done using the one vs. one approach. The results are shown in Figure 10 and Figure 11 where the scatter plot and the dependency on the regularization parameter is displayed, respectively. In the hyperparameter selection phase, the best results were obtained for C equal to 20, but we have found that the impact of this parameter is rather low as shown in Figure 11.

Furthermore, also in this case, the accuracy on the test set was above 0.99, the achievable performance being comparable to the *k*NN classifier. The advantage here is that the whole set of training samples need not be stored for the prediction, but on the other hand, due to the multi-class generalization there are in fact multiple component models forming the final prediction. The absolute error values are displayed in Table 2, showing that the model performance is comparable to *k*NN.

### 4.3. Random Forest

**Figure 12:**
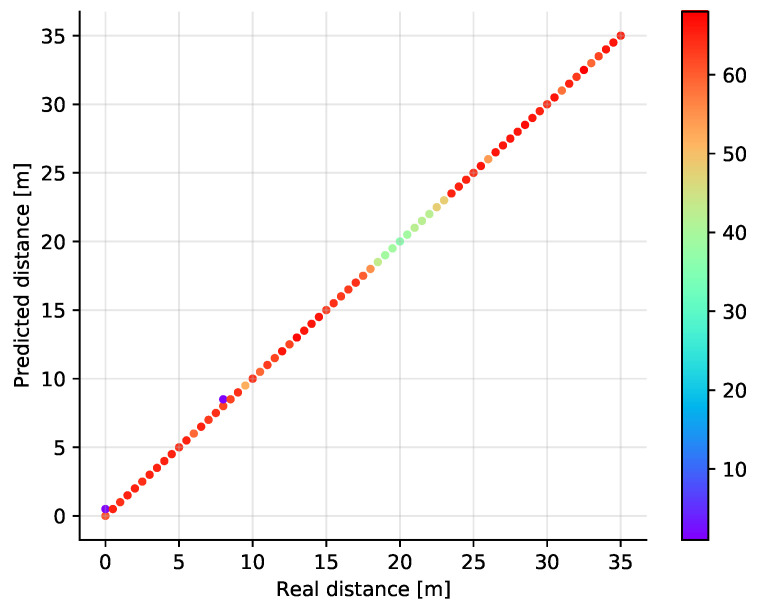
Scatter plot showing the predicted versus the real distances in a single dimension with the Random Forest classifier with the number of trees equal to 160.

The Random Forest classifier is based on an ensemble of decision trees which classify the given sample by following a set of conditions determined in the training phase. We have experimented with this approach, varying the number of trees (decision elements) in the structure between 10 and 200. The results show that the Random Forest classifier is able to classify the dataset accurately (see Figure 12). Actually, the results achieved are among the best overall, with the accuracy over 99.9% in all cases in which the number of estimators is above 40. The best results in the validation phase were obtained when the number of estimators was 180. Interestingly, in the cases where the model did not classify the position correctly, it selected the closest point so the maximal position error is 0.5 m.

### 4.4. Multi-Layer Perceptron

All the ML approaches presented in the previous subsections have shown their potential to serve as very good location estimators in our scenario. Still, the Artificial Neural Network paradigm has found to be a good application in many classification problems. Thus, we have also applied a simple ANN in the form of a Multi-Layer Perceptron (MLP) with multiple hidden layers activated by the Rectified Linear Unit (ReLU) activation function and the logistic function as the activation of the output layer.

**Figure 13:**
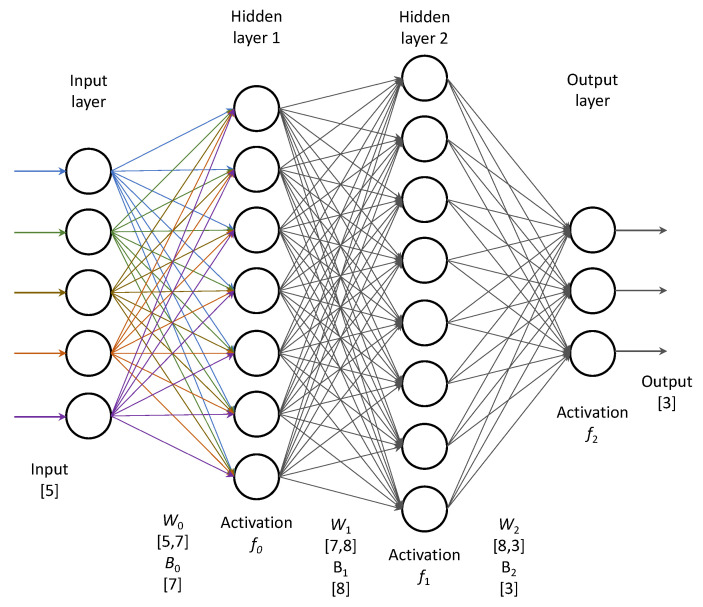
A sample structure of a Multi-Layer Perceptron with five inputs, two hidden layers and three outputs.

The network takes as many inputs as there are input parameters and has as many outputs as there are possible output classes. The basic structure of the MLP with two hidden layers is shown in Figure 13. In the figure, we show the dimension of weight matrices and bias vectors at each layer of the network for the case of two hidden layers. In a general case, for *m* input features, *n* outputs (classes) and *o*, *p* neurons in two hidden layers, the network will have (n·o+o+o·p+p·m+m) parameters to learn, in the case shown in the figure the number of parameters for this simple network is 133 and grows with the increase of the number of neurons in the hidden layers.

In the hyperparameter optimization, we have varied the number of hidden layers (up to 3) and the number of neurons in each layer (varying exponentially from 5 up to 1280). We have found that there is no increase of performance when the third hidden layer is added, so the final model had two hidden layers with 640 and 320 neurons in the hidden layers, respectively.

**Figure 14:**
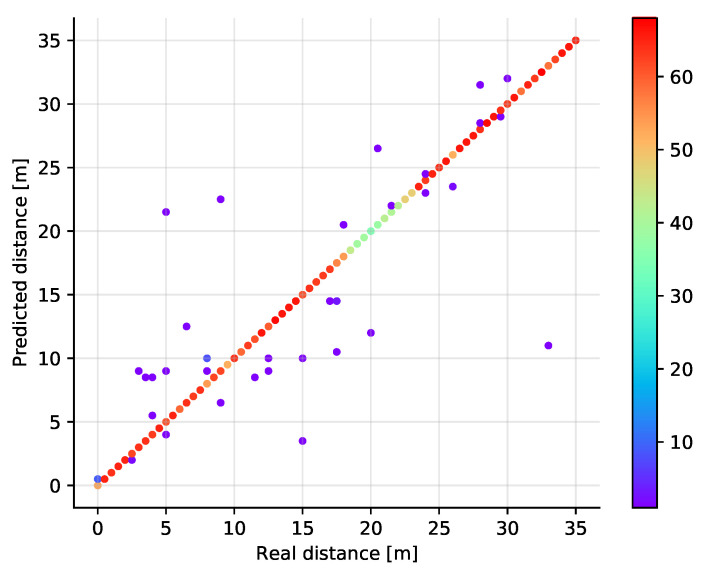
Scatter plot showing the predicted versus the real distances in a single dimension using the Multi Layer Perceptron with the two hidden layers containing 640 and 320 neurons.

As obvious from the results (see Figure 14), the MLP has had inferior performance to all solutions described above. In no case did the classification accuracy on the test set reach over 0.98. Given the complexity of the model and the performance of other techniques, the MLP classifier is not suitable for the given problem.

## 5. Two-Dimensional Positioning

**Figure 15:**
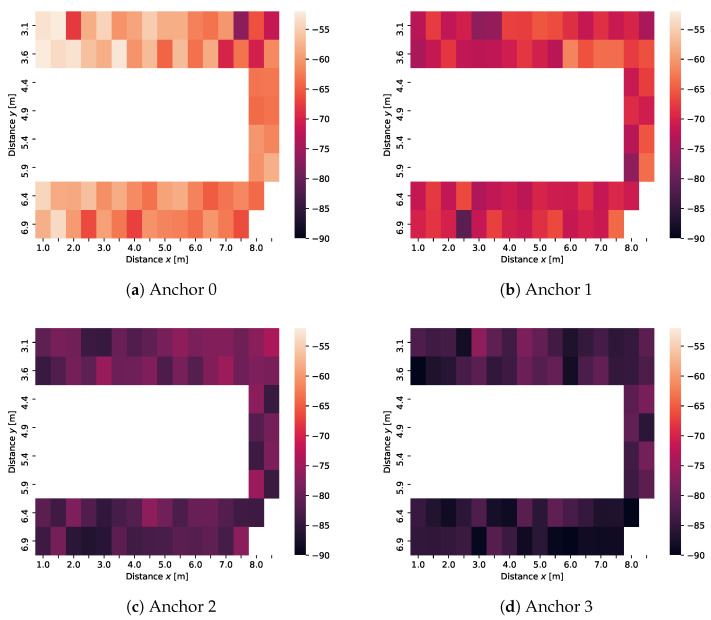
Mean values of received signal strengths (RSSs) at four anchor location when the transmitter is the moving tag in a two-dimensional position grid. All values (see legends) are in units dBm. For each location, the mean of all received measurement samples is displayed, Channel 37.

The problem gets more complicated when we aim for two-dimensional positioning with the same set of anchors, allowing the tag to travel also in the perpendicular direction through laboratory rooms next to the corridor. Thus, we aim to estimate location along two axes: parallel (*X*) and perpendicular (*Y*) to the axis along which the anchors are located in the corridor. A visualization of measured data for one laboratory is available in Figure 15 where each of the subplots represents signals received on one of the four anchors. The visualization corresponds to the rightmost room in Figure 3. We only consider one frequency (channel 37) in the visualisations, the color of each area representing the mean of all measured values for the particular position.

Obviously, anchor 0 was the closest to the room with the highest RSS values, anchor 3 was the farthest. The locations have a different coordinate system compared to the one dimensional case in Section 4, which means that the required models have to be re-trained. We have assigned each location a unique id made up from the (*X*) and (*Y*) coordinates. In total, there are 300 possible locations in the two dimensional model—213 valid locations correspond to the corridor, 18 and 69 correspond to the two laboratories in Figure 3, respectively. In the end, this means the model needs to classify each sample into one of 300 classes.

### 5.1. kNN Classifier

For the case of the *k* Nearest Neighbors classifier we have tested a variable number of neighbors also in the two-dimensional case. Similar to the one-dimensional case, the classifier works best with rather few neighbors, in this case the best results were obtained for k=2 in the 5-fold cross validation. In this case, it is possible to achieve the correct classification in more than 99% of cases as shown in Table 3.

**Figure 16:**
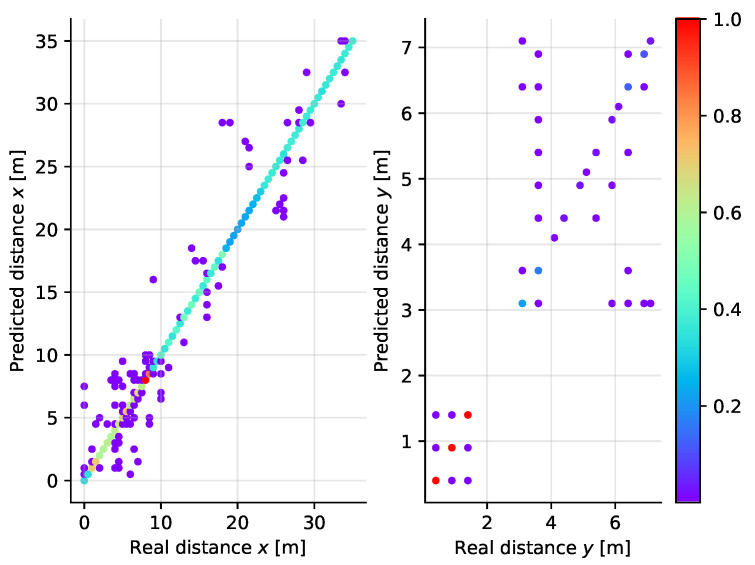
Scatter plot diagram of the real and predicted distance along the *X* (**left**) and *Y* (**right**) axes for the *k*NN classifier applied to two-dimensional localization. The colour of the points corresponds to normalized count of samples at a given position of the plot.

As shown in Figure 16, the majority of points have been classified correctly. This plot, for clarity, uses a color scheme to address the number of occurences for each point. In order to align the scale of the two subplots, the values are normalized with respect to the maximum. We can observe from the figure that the errors appear mainly in the *y* coordinate. One reason is that the number of valid measurements is lower at the locations in the laboratory rooms than at the corridor locations. The second reason is that signals received in the rooms are subject to higher attenuation due to wall penetration which causes the signal levels at distant anchors to be rather low. The third reason is the geometry of the experiment in which the distance differences from anchors in the *Y* direction are lower than the distance differences in the *X* direction.

In the two-dimensional case, we can obtain the absolute spatial distances by evaluating the errors in the *X* and in the *Y* coordinate for each sample in the test set. Then, the location error can simply be calculated using the Pythagorean theorem. In this way, we can obtain the summary statistics of the maximal error distance and the mean error distance as shown in Table 3. In this case the errors are also discrete valued, but since diagonal errors are also possible, they will not in general be multiples of 0.5 m.

### 5.2. SVM Classifier

**Figure 17:**
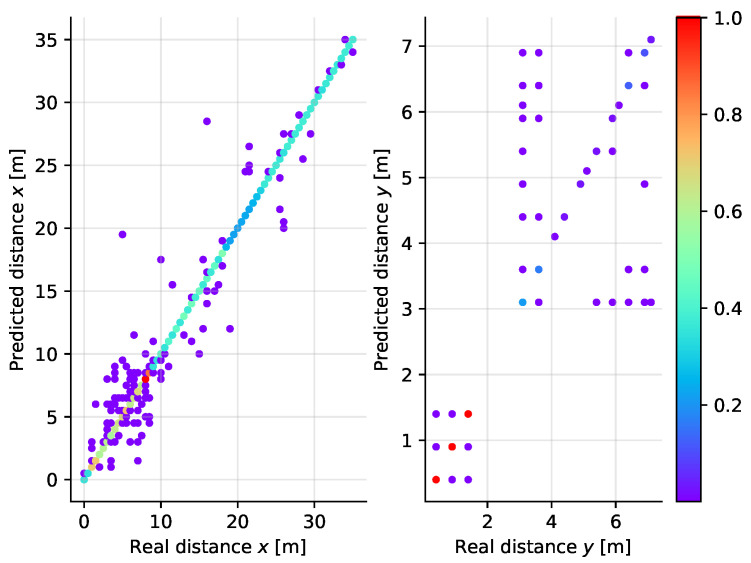
Scatter plot diagram of the real and predicted distance along the *X* (**left**) and *Y* (**right**) axes for the SVM classifier applied to two-dimensional localization.

In the SVM classifier, we have found that the impact of the regularization parameter is stronger in the two-dimensional case than in simpler classification along a single axis. Larger values of the parameter *C* tend to improve the performance of the model, although even for C=38 the performance on the test set is slightly worse than that experienced for *k*NN. Having in mind that the increased regularization parameter strongly impacts the learning time, the value 38 has been the maximum tested with the accuracy of classification reaching 99%. The scatter plot diagram for the SVM classifier in the two-dimensional case is shown in Figure 17. Clearly, the spread of values is comparable to the case of Figure 16.

### 5.3. Random Forest Classifier

**Figure 18:**
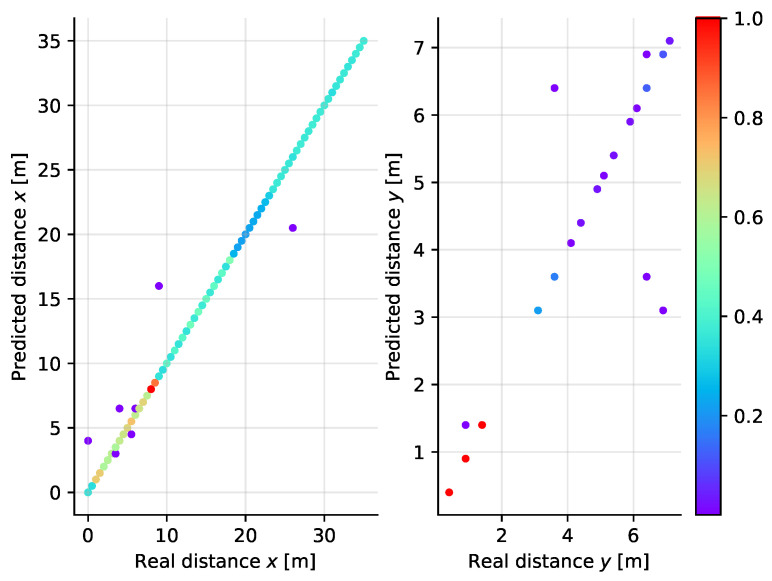
Scatter plot diagram of the real and predicted distance along the *X* (**left**) and *Y* (**right**) axes for the Random Forest classifier applied to two-dimensional localization.

The Random Forest (RF) classifier performs superior to all other algorithms in this case (see Figure 18) as well as in the simpler one-dimensional scenario. Recall that in the one-dimensional case, RF was among the best options, however in the two-dimensional scenario in which the problem is more complex the performance of the algorithm is much more strongly visible.

Varying the number of predictors in the RF ensemble, we see that the best performance is achieved when the number of trees is 240, which is the largest value we have tested to keep the complexity of the model maintainable. Even in this complex case with 300 output classes, the accuracy of the model on the test set is over 99.9%.

### 5.4. Multi-Layer Perceptron

**Figure 19:**
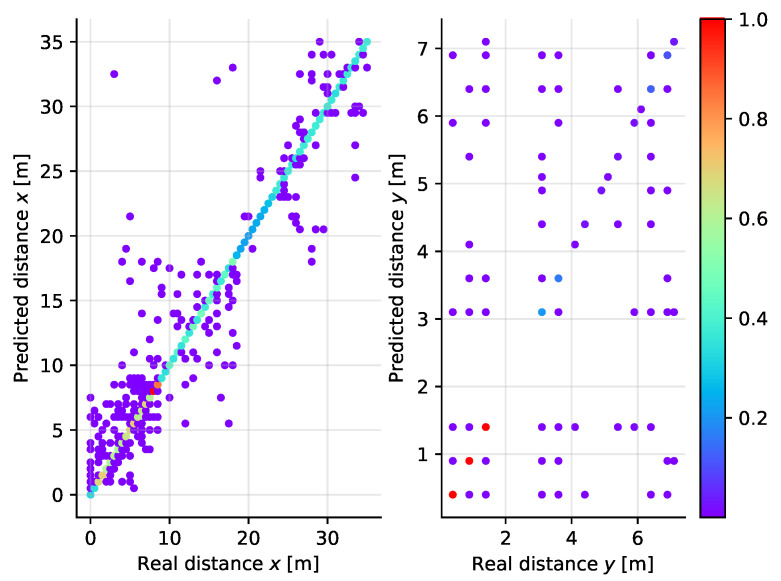
Scatter plot diagram of the real and predicted distance along the *X* (**left**) and *Y* (**right**) axes for the Multi-Layer Perceptron classifier applied to two-dimensional localization.

In this case we have taken networks with up to three hidden layers consisting of up to 1280 neurons as the candidate architectures. The best results have been achieved with 1280 + 1280 neurons in the hidden layers, which means that for the larger dataset (compared to the one-dimensional case) a more complex network is required. Still, given the complexity of the model and the computational complexity of the training, the results achieved (see Figure 19) are worse compared to the other cases—the accuracy over all points (*X* and *Y* correct at the same time) is 97.6%. The neural network is also prone to highly distant misclassifications as in the worst case the spatial distance between the correct and predicted class was almost 30 m.

## 6. Summary and Conclusions

To compare the performance of the different ML techniques in both the one-dimensional and the two-dimensional localization scenario, we provide an overview of test set accuracy in Table 2 and Table 3, respectively. In these tables, we provide three values for each approach. The accuracy achieved in the test set is computed as the ratio of correctly classified positions to the whole number of samples in the test set. The maximal error is the spatial distance of the farthest misclassified position from the correct location and the mean error is the average spatial distance of all predictions.

From the results it is obvious that Bluetooth Low Energy can well be used for RSS-based localization although the received signal levels tend to fluctuate over time at any given position. Machine learning algorithms provide the opportunity to achieve very high accuracy and low errors as particularly seen in the case of Random Forest classifier.

In this work, we have provided an overview of the possible approaches to improving the performance of the fingerprinting technique using the supervised machine learning paradigm. We have defined a laboratory environment and performed a measurement campaign to collect a representative number of samples for localization based on RSS fingerprints in the radio environment of Bluetooth Low Energy. The collected data are freely available, ready to be re-used in further work also by other researchers.

The complexity of the different models is well illustrated by the disk size needed to store the model for inference. Considering the hyperparameter adjustment and the one dimensional scenario as described in Section 4 for example, the largest disk footprint has the Random Forest classifier (175 MB), which is the best performing algorithm. This may limit the usability of this approach in practical applications. The size of the other models is comparable, with *k*NN requiring 3.8 MB, Multi-Layer Perceptron using 2.5 MB and the SVM model using 6.9 MB.

The results achieved in the RSS fingerprinting employing machine learning show that with proper selection of algorithm and with a sufficient number of samples for training, the localization can be very accurate. The accuracy achieved for three of the best performing algorithms is over 99%. Among these, the *k* Nearest Neighbor classifier has slightly lower performance and comes with the requirement to store the learning samples (neighbors) for the inference phase. The Support Vector Machine, on the other hand, requires a complex model due to the high number of output classes. The Random Forest classifier scored best in our experiment and is seen as a very promising technique in this specific application.

The work presented in this paper evaluates the usage of machine learning algorithms for positioning in fixed conditions, where not many external factors are expected to influence the working of the position estimation. As a follow-up work, we aim to extend the data-set and the models to evaluate how factors such as presence of humans, changes in furniture position or imprecise ground truth measurement deteriorate the obtained results.

## Figures and Tables

**Table 1:** Summary of literature considering Machine Learning for localization algorithm improvement and data fusion in the context of RSS localization.

Classifier or Regessor	RSS Fingerprint. Improvement	Fusion of RSS and Other Information
Support Vector Machine	[21,26]	[28]
Random Forest	[24]	[28]
Logistic Regression	[21]	
*k* Nearest Neighbors	[26,29,32]	[22,29]
Sum of Squared Differences	[23]	
Particle Markov Model		[29]
Polynomial Regression		[30]
Naive Bayes	[24]	
Artificial Neural Network	[25,27]	

**Table 2:** Summary of classifier performance in the one-dimensional classification problem.

Classifier	Test Set Accuracy	Max. Error	Mean Error
*k*NN (1 neighbor)	0.995813	4.5 m	0.00697 m
SVM	0.996046	5.0 m	0.00837 m
Rand. forest	0.999534	0.5 m	0.00023 m
MLP	0.970697	22.0 m	0.04162 m

**Table 3:** Summary of classifier performance in the two-dimensional classification problem.

Classifier	Test Set Accuracy	Max. Error	Mean Error
*k*NN	0.992174	10.51 m	0.01872 m
SVM	0.991553	14.50 m	0.02176 m
Rand. forest	0.999503	7.01 m	0.00188 m
MLP	0.975965	29.54 m	0.08150 m

## Data Availability

Links on datasets are available in the article.

## References

[1] Zafari F., Gkelias A., Leung K.K. (2019). A Survey of Indoor Localization Systems and Technologies. IEEE Commun. Surv. Tutor..

[2] Al-Ammar M.A., Alhadhrami S., Al-Salman A., Alarifi A., Al-Khalifa H.S., Alnafessah A., Alsaleh M. Comparative Survey of Indoor Positioning Technologies, Techniques, and Algorithms. Proceedings of the 2014 International Conference on Cyberworlds.

[3] Stavrou V., Bardaki C., Papakyriakopoulos D., Pramatari K. (2019). An Ensemble Filter for Indoor Positioning in a Retail Store Using Bluetooth Low Energy Beacons. Sensors.

[4] Xiao J., Zhou Z., Yi Y., Ni L.M. (2016). A survey on wireless indoor localization from the device perspective. ACM Comput. Surv. CSUR.

[5] Yassin A., Nasser Y., Awad M., Al-Dubai A., Liu R., Yuen C., Raulefs R., Aboutanios E. (2017). Recent Advances in Indoor Localization: A Survey on Theoretical Approaches and Applications. IEEE Commun. Surv. Tutor..

[6] Baert M., Rossey J., Shahid A., Hoebeke J. (2018). The Bluetooth mesh standard: An overview and experimental evaluation. Sensors.

[7] Yang J., Poellabauer C., Mitra P., Neubecker C. (2020). Beyond beaconing: Emerging applications and challenges of BLE. Ad Hoc Netw..

[8] Kriz P., Maly F., Kozel T. (2016). Improving indoor localization using Bluetooth low energy beacons. Mob. Inf. Syst..

[9] Neburka J., Tlamsa Z., Benes V., Polak L., Kaller O., Bolecek L., Sebesta J., Kratochvil T. Study of the performance of RSSI based Bluetooth Smart indoor positioning. Proceedings of the 2016 26th International Conference Radioelektronika (RADIOELEKTRONIKA).

[10] Pelant J., Tlamsa Z., Benes V., Polak L., Kaller O., Bolecek L., Kufa J., Sebesta J., Kratochvil T. BLE device indoor localization based on RSS fingerprinting mapped by propagation modes. Proceedings of the 2017 27th International Conference Radioelektronika (RADIOELEKTRONIKA).

[11] Rozum S., Sebesta J. SIMO RSS measurement in Bluetooth low power indoor positioning system. Proceedings of the 2018 28th International Conference Radioelektronika.

[12] Rozum S., Kufa J., Polak L. Bluetooth Low Power Portable Indoor Positioning System Using SIMO Approach. Proceedings of the 2019 42nd International Conference on Telecommunications and Signal Processing (TSP).

[13] Li G., Geng E., Ye Z., Xu Y., Lin J., Pang Y. (2018). Indoor positioning algorithm based on the improved RSSI distance model. Sensors.

[14] Giuliano R., Cardarilli G.C., Cesarini C., Di Nunzio L., Fallucchi F., Fazzolari R., Mazzenga F., Re M., Vizzarri A. (2020). Indoor localization system based on Bluetooth low energy for museum applications. Electronics.

[15] Atashi M., Malekzadeh P., Salimibeni M., Hajiakhondi-Meybodi Z., Plataniotis K.N., Mohammadi A. Orientation-matched multiple modeling for RSSI-based indoor localization via BLE sensors. Proceedings of the 2020 28th European Signal Processing Conference (EUSIPCO).

[16] Ng P.C., She J., Rong R. (2020). Compressive RF Fingerprint Acquisition and Broadcasting for Dense BLE Networks. IEEE Trans. Mob. Comput..

[17] Heyn R., Kuhn M., Schulten H., Dumphart G., Zwyssig J., Trosch F., Wittneben A. User Tracking for Access Control with Bluetooth Low Energy. Proceedings of the 2019 IEEE 89th Vehicular Technology Conference (VTC2019-Spring).

[18] Wisanmongkol J., Klinkusoom L., Sanpechuda T., Kovavisaruch L., Kaemarungsi K. Multipath Mitigation for RSSI-Based Bluetooth Low Energy Localization. Proceedings of the 2019 19th International Symposium on Communications and Information Technologies (ISCIT).

[19] Sun X., Ai H., Tao J., Hu T., Cheng Y. (2021). BERT-ADLOC: A secure crowdsourced indoor localization system based on BLE fingerprints. Appl. Soft Comput..

[20] Abed A., Abdel-Qader I. (2019). RSS-Fingerprint Dimensionality Reduction for Multiple Service Set Identifier-Based Indoor Positioning Systems. Appl. Sci..

[21] Sthapit P., Gang H.S., Pyun J.Y. Bluetooth Based Indoor Positioning Using Machine Learning Algorithms. Proceedings of the 2018 IEEE International Conference on Consumer Electronics-Asia (ICCE-Asia).

[22] Duong N.S., Dinh T.M. Indoor Localization with lightweight RSS Fingerprint using BLE iBeacon on iOS platform. Proceedings of the 2019 19th International Symposium on Communications and Information Technologies (ISCIT).

[23] Kajioka S., Mori T., Uchiya T., Takumi I., Matsuo H. Experiment of indoor position presumption based on RSSI of Bluetooth LE beacon. Proceedings of the 2014 IEEE 3rd Global Conference on Consumer Electronics (GCCE).

[24] Campaña F., Pinargote A., Domínguez F., Peláez E. Towards an indoor navigation system using Bluetooth Low Energy Beacons. Proceedings of the 2017 IEEE Second Ecuador Technical Chapters Meeting (ETCM).

[25] Iqbal Z., Luo D., Henry P., Kazemifar S., Rozario T., Yan Y., Westover K., Lu W., Nguyen D., Long T. (2018). Accurate real time localization tracking in a clinical environment using Bluetooth Low Energy and deep learning. PLoS ONE.

[26] Lovon-Melgarejo J., Castillo-Cara M., Orozco-Barbosa L., García-Varea I. Supervised learning algorithms for indoor localization fingerprinting using BLE4.0 beacons. Proceedings of the 2017 IEEE Latin American Conference on Computational Intelligence (LA-CCI).

[27] Jondhale S.R., Deshpande R.S. (2019). GRNN and KF framework based real time target tracking using PSOC BLE and smartphone. Ad Hoc Netw..

[28] Takayama T., Umezawa T., Komuro N., Osawa N. An Indoor Positioning Method Based on Regression Models with Compound Location Fingerprints. Proceedings of the 2018 Ubiquitous Positioning, Indoor Navigation and Location-Based Services (UPINLBS).

[29] Sou S.I., Lin W.H., Lan K.C., Lin C.S. (2019). Indoor Location Learning Over Wireless Fingerprinting System with Particle Markov Chain Model. IEEE Access.

[30] Zhuang Y., Yang J., Li Y., Qi L., El-Sheimy N. (2016). Smartphone-Based Indoor Localization with Bluetooth Low Energy Beacons. Sensors.

[31] Theodoridis S., Pikrakis A., Koutroumbas K., Cavouras D. (2010). Introduction to Pattern Recognition: A MATLAB Approach.

[32] Duong S.N., Trinh A.V.T., Dinh T.M. Bluetooth Low Energy Based Indoor Positioning on iOS Platform. Proceedings of the 2018 IEEE 12th International Symposium on Embedded Multicore/Many-core Systems-on-Chip (MCSoC).

[33] Liu H., Darabi H., Banerjee P., Liu J. (2007). Survey of Wireless Indoor Positioning Techniques and Systems. IEEE Trans. Syst. Man Cybern. Part C Appl. Rev..

[34] Telegies (2010). ETRX35x ZigBee Modules.

[35] Laird Connectivity (2016). BL652 Series Bluetooth v5.

[36] STMicroelectronics (2015). ARM-Based Microcontroller.

[37] PulseLarsen Electronics (2007). Wireless External Antenna for 2.4 GHz Applications.

[38] Jung Y. (2017). Multiple predicting K-fold cross-validation for model selection. J. Nonparametr. Stat..

[39] Fong-Mata M.B., García-Guerrero E.E., Mejía-Medina D.A., López-Bonilla O.R., Villarreal-Gómez L.J., Zamora-Arellano F., López-Mancilla D., Inzunza-González E. (2020). An Artificial Neural Network Approach and a Data Augmentation Algorithm to Systematize the Diagnosis of Deep-Vein Thrombosis by Using Wells’ Criteria. Electronics.

[40] Tian X., Wang M., Li W., Jiang B., Xu D., Wang X., Xu J. (2017). Improve accuracy of fingerprinting localization with temporal correlation of the RSS. IEEE Trans. Mob. Comput..

[41] Hoang M.T., Zhu Y., Yuen B., Reese T., Dong X., Lu T., Westendorp R., Xie M. (2018). A soft range limited K-nearest neighbors algorithm for indoor localization enhancement. IEEE Sens. J..

[42] Pedregosa F., Varoquaux G., Gramfort A., Michel V., Thirion B., Grisel O., Blondel M., Prettenhofer P., Weiss R., Dubourg V. (2011). Scikit-learn: Machine Learning in Python. J. Mach. Learn. Res..

